# Acquired equivalence associative learning in GTC epileptic patients: experimental and computational study

**DOI:** 10.3389/fncel.2015.00418

**Published:** 2015-10-27

**Authors:** Radwa Khalil, Noha Abo Elfetoh, Marie Z. Moftah, Eman M. Khedr

**Affiliations:** ^1^Department of Cognitive Biology, Otto-von-Guericke UniversitätMagdeburg, Germany; ^2^Department of Developmental Physiology, Institute of Physiology, Otto-von-Guericke UniversitätMagdeburg, Germany; ^3^IMN - Institut des Maladies Neurodégénératives, University of BordeauxBordeaux, France; ^4^Department of Neurology, Faculty of Medicine, Assiut UniversityAssiut, Egypt; ^5^Department of Zoology, Faculty of Science, Alexandria UniversityAlexandria, Egypt

**Keywords:** generalized tonic clonic epilepsy, cognitive impairment, acquired equivalence associative learning task, basal ganglia, hippocampus, connectionist model

## Abstract

Previous cognitive behavioral studies based on Acquired Equivalence Associative learning Task (AEALT) showed a strong relation between hippocampus and basal ganglia in associative learning. However, experimental behavioral studies of patients with Generalized Tonic Clonic (GTC) epilepsy remained sparse. The aim of the present study is to integrate a classical behavioral cognitive analysis with a computational model approach to investigate cognitive associative learning impairments in patients with GTC epilepsy. We measured the accuracy of associative learning response performance in five GTC epileptic patients and five control subjects by using AEALT, all subjects were matched in age and gender. We ran the task using E-Prime, a neuropsychological software program, and SPSS for data statistical analysis. We tested whether GTC epileptic patients would have different learning performance than normal subjects, based on the degree and the location of impairment either in basal ganglia and/or hippocampus. With the number of patients that was available, our behavioral analysis showed no remarkable differences in learning performance of GTC patients as compared to their control subjects, both in the transfer and acquisition phases. In parallel, our simulation results confirmed strong connection and interaction between hippocampus and basal ganglia in our GTC and their control subjects. Nevertheless, the differences in neural firing rate of the connectionist model and weight update of basal ganglia were not significantly different between GTC and control subjects. Therefore, the behavioral analysis and the simulation data provided the same result, thus indicating that the computational model is likely to predict cognitive outcomes.

## Introduction

Acquired Equivalence Associative learning Task (AEALT) is a psychological cognitive task for associative learning assessment (Moustafa et al., 2000; Myers et al., 2003; Herzallah et al., 2010; Moustafa and Gluck, 2011). Importantly, several behavioral and experimental studies based on AEALT provided evidence for a strong interaction between hippocampus and basal ganglia in associative learning (Honey and Hall, 1991; Hall et al., 1993; Moustafa et al., 2000; Coutureau et al., 2002; Myers et al., 2003; Bodi et al., 2009). Moreover, it has been used to assess cognitive impairment in several brain regions, in particular the temporal lobe including the hippocampus and the frontal lobe including the basal ganglia (Moustafa et al., 2000; Myers et al., 2003; Herzallah et al., 2010; Moustafa and Gluck, 2011). Additionally, various cognitive profiles of different neurological and neuropsychological disorders have been also investigated using AEALT (Moustafa et al., 2000; Myers et al., 2003; Herzallah et al., 2010; Moustafa and Gluck, 2011). However, an AEALT-based behavioral study of Generalized Tonic Clonic (GTC) epilepsy remains sparse. In the present study; we applied AEALT (Moustafa et al., 2000; Myers et al., 2003; Herzallah et al., 2010; Moustafa and Gluck, 2011), to investigate cognitive impairments in GTC epileptic patients using combined experimental behavioral and computational approaches. Our main focus is to show how such computational approach could reproduce, at the functional level, the result obtained from the experimental analysis, thus validating the simulation protocol. Thus, our main purpose does not purely focusing on presenting a separate computational-theoretical study neither a segregated clinical case report independently but rather to validate the simulation approach by comparing our simulated results with the experimental behavioral results as measured in GTC-epileptic patients and controls. In other words, our attention was to test how our computational model (if correctly fed with experimental data from representative patients) could mimic the results of cognitive behavioral test.

Our neurobiological hypothesis is based on the fact that GTC is characterized by generalized seizures which invade most brain regions, in particular the temporal lobe also involving hippocampus and the frontal lobe, including basal ganglia. In parallel, the theoretical hypothesis of AEALT (Moustafa et al., 2000; Myers et al., 2003; Herzallah et al., 2010; Moustafa and Gluck, 2011), which is one of the classical cognitive learning tasks to measure the associative learning performance, relied on the idea that one region is associated with the acquisition while the other one with the transfer phase. This hypothesis suggests that this category of learning might be altered in GTC patients. Therefore, we measured associative learning performances in a group of GTC patients and in their matched healthy controls. For this purpose, AEALT (Moustafa et al., 2000; Myers et al., 2003; Herzallah et al., 2010; Moustafa and Gluck, 2011), was used for describing the connection between basal ganglia and hippocampus and their interaction in acquisition and transfer phases (Moustafa et al., 2000; Myers et al., 2003; Herzallah et al., 2010; Moustafa and Gluck, 2011). It is often difficult to identify the appropriate level of modeling for a particular problem and it is a frequent mistake to assume that a highly detailed model is necessarily superior. In the present study, we did not try to set a pure abstract cognitive model but, rather to feed this model with the output of the subjects' data. Accordingly, we used the actual experimental data collected from our groups, GTC-epileptic patients and controls, after performing AEALT (Moustafa et al., 2000; Myers et al., 2003; Herzallah et al., 2010; Moustafa and Gluck, 2011), whereas these actual data represented the input values for our simulated model. In other word, the behavioral task, AEALT (Moustafa et al., 2000; Myers et al., 2003; Herzallah et al., 2010; Moustafa and Gluck, 2011), served as a read out for cognitive functions and documented the associative learning. In addition, the modeling approach explored the role of the temporal and frontal lobe, and more particularly of the hippocampus and basal ganglia in the AEALT associative learning task.

## Methodology

### Experimental behavioral study

#### Description of acquired equivalence associative learning task (AEALT)

**Figure 1:**
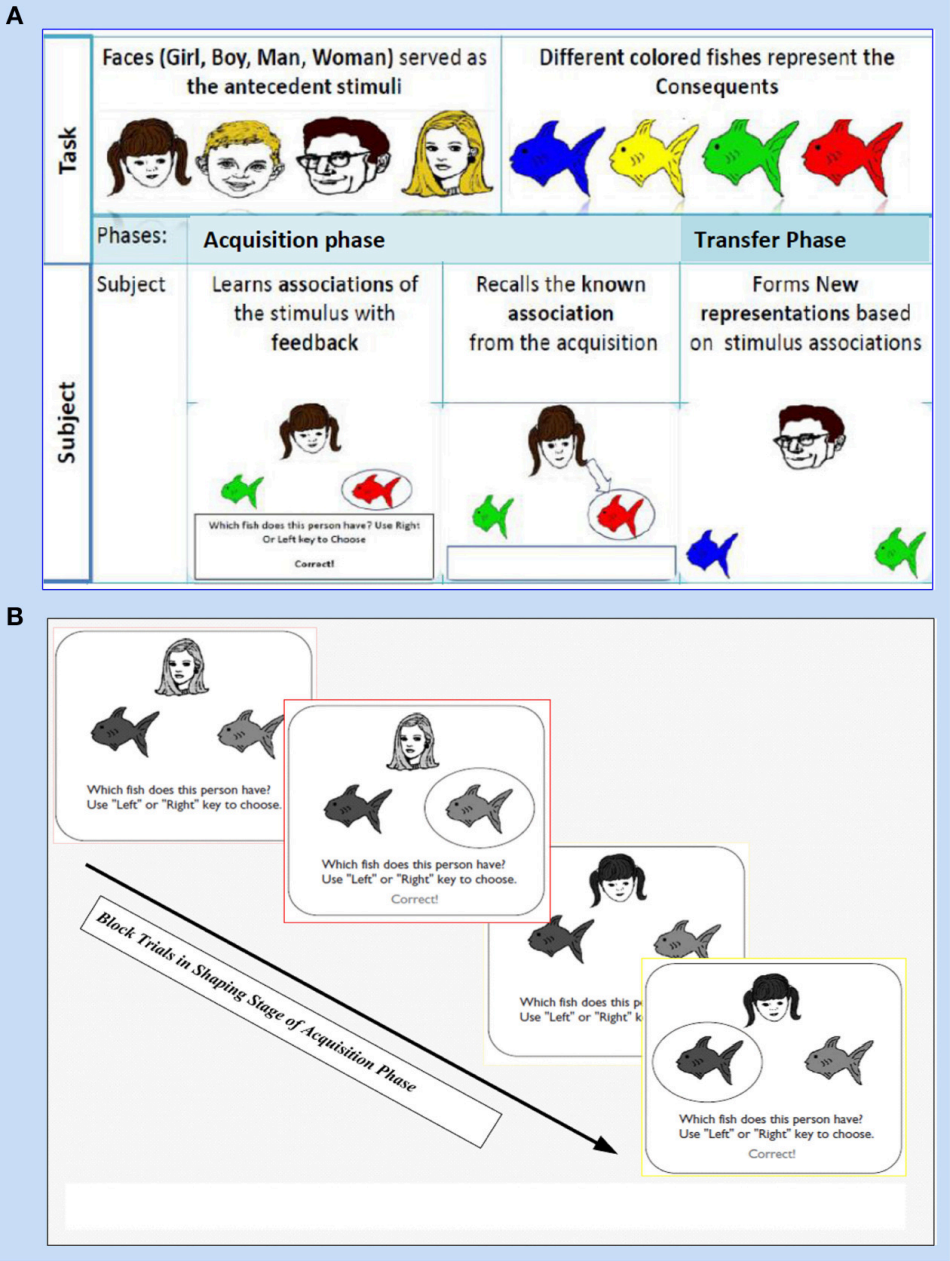
**It shows Acquired Equivalence Associative learning Task (AEALT); this task is (adapted from Moustafa et al., 2000; Myers et al., 2003; Herzallah et al., 2010; Moustafa and Gluck, 2011)**. **(A)** Represents the phases of AEALT; acquisition and transfer phases (see task description, Methodology Section), whilst **(B)** is a screen snapshot represents experimental trials in an early stage of acquisition phase which is considered as shaping and training stage. In this latter stage, the stimulus is represented as a human face appearing on the screen and subsequently, the subject responds by choosing one of the colored fish either by clicking left or right, only then, the correct feedback is given (see task description, Methodology Section).

This task (adapted from Moustafa et al., 2000; Myers et al., 2003; Herzallah et al., 2010; Moustafa and Gluck, 2011) comprises of two sorts of stimuli; antecedent and consequent (see Figure 1). On one hand, the antecedent stimuli are represented by four drawings of human faces show distinctive ages, youthful and adult human, and genders, females and males; i.e., woman, boy, man, and girl. On the other hand, four drawings of fish with several colors, red, orange, purple, and pink, are related to the consequents. In general, this task incorporates two phases: (i) The acquisition phase, and (ii) The transfer phase. During the acquisition phase, both stimuli are associated with each other; the antecedent and the consequent one. Each subject has to associate a particular human face with a specific colored fish, by clicking on the keyboard arrow either right or left (see Figure 1A). In the training stage, two antecedent stimuli A1 and A2, are associated with the same consequent stimulus X1, while two antecedent stimuli B1 and B2 are associated with consequent Y1, i.e., subject has to guess which face is related to which fish. Immediately, the selected fish is circled and the correct feedback is given (Figure 1B). Next, A1 is associated with a new consequent X2 while B1 is associated with another novel consequent Y2. (ii) Finally, a transfer phase tests whether patients would show acquired equivalence and associate A2 with X2, and B2 with Y2, even though these particular stimulus pairings had never been trained. This phase corresponds to a new association that could be formed according to the principle of acquired equivalence.

#### Subjects

##### Neuropsychological background

We tested five subjects with GTC epilepsy (*n* = 5, Women, over 19 year old) and five controls. All subjects were matched on gender, age and education. Importantly, they were all matched in terms of educational levels and seizure onset. All GTC patients were on treatment since early age, between 12 and 16 years old. Their ages were above 19 and up to 41 years old and their controls are matching them. Additionally, we screened them for the absence of any neurological or psychiatric disorders that could interfere with epileptic symptoms. We allowed our subjects to perform AEALT (Moustafa et al., 2000; Myers et al., 2003; Herzallah et al., 2010; Moustafa and Gluck, 2011), after they passed the average scores of several Intelligence Quotient (IQ) subtests, which represented Wechsler Intelligence Scale for Children (WISC). The original WISC (Wechsler, 1949) is an adaptation of several of the subtests used for the Wechsler Bellevue Intelligence Scale (Wechsler, 1939), which also proposed several specific subtests. These subtests were organized into Verbal and Performance scales, and provided scores for Verbal (VIQ), Performance (PIQ), and Full Scale IQ (FSIQ). We only selected the GTC patients who had a relatively high IQ score to ensure that they were able to go through the AEALT (Moustafa et al., 2000; Myers et al., 2003; Herzallah et al., 2010; Moustafa and Gluck, 2011). That was one of the reasons that made us ending up with small sample size, only five patients and five healthy controls. These averages of scores were in the following ranges (IQ = 60–79, VIQ = 62–80, PIQ = 63–77), we observed that the level of IQ and its subtests decreased with the increasing the age.

##### Ethical approval for subjects' participation

We performed this experimental behavioral study in accordance with the declaration of the medical university, after getting the official approval by *Assiut Medical hospitals, Assiut, Egypt* whereas all participant subjects gave written informed consent.

##### Statistical analyses

After task performance, we saved patients' data and imported them directly into the SPSS, statistical software for analysis of the learning performance. Then, we performed a Mann–Whitney test to assess the significance level of learning response accuracy between GTC epileptic patients and their healthy controls.

### Computational study

#### Computational model structure and description

**Figure 2:**
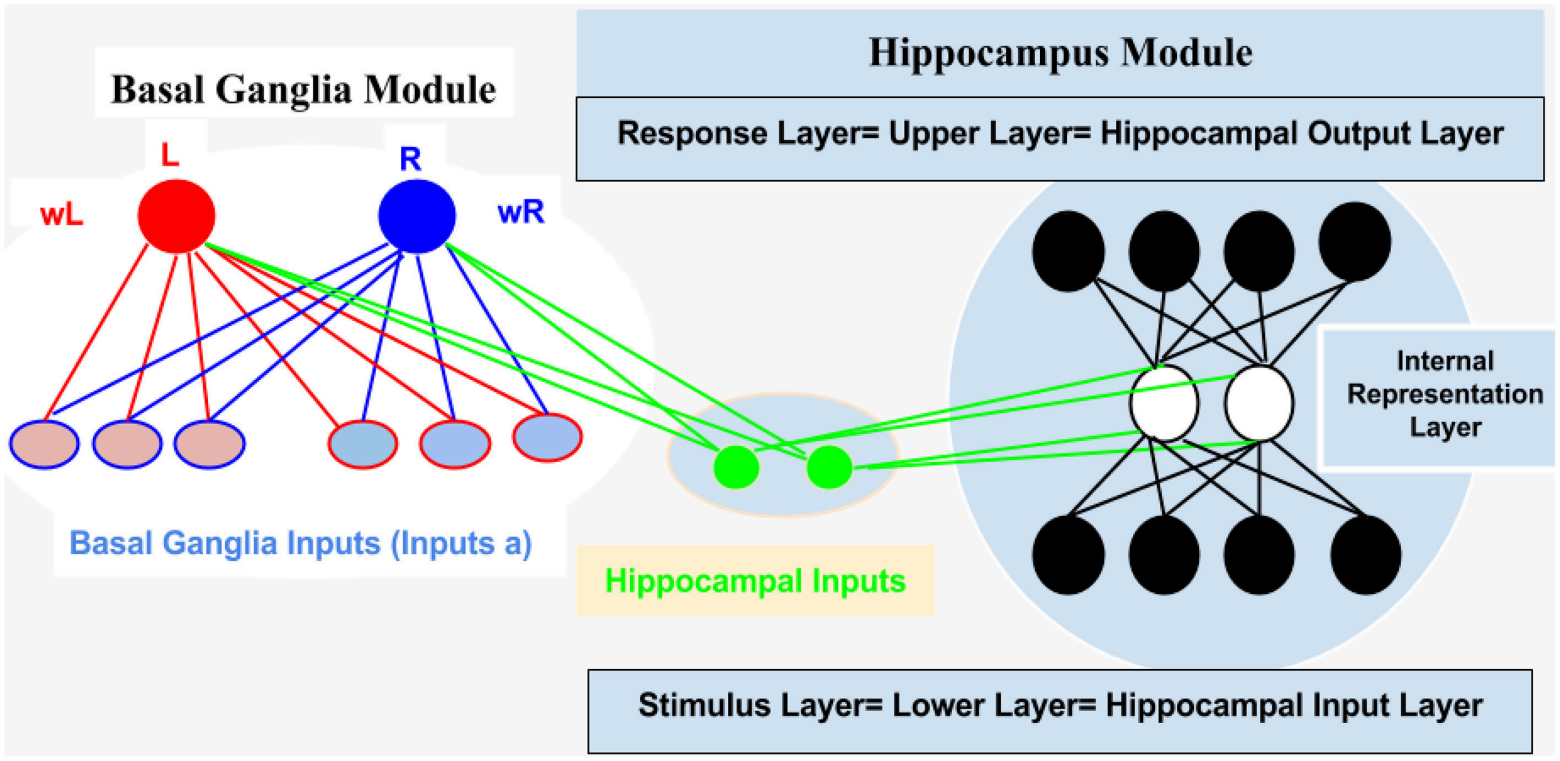
**It represents structural description of the connectionist model, see text for illustration, Methodology Section**.

For computational and simulation study, we introduced an abstract connectionist model (for similar study; see Gluck and Rumelhart, 1990; Dayan and Abbott, 2001; Chapter 9 Classical Conditioning and Reinforcement Learning, and Moustafa et al., 2009), which is based on the structural analysis of AEALT (Moustafa et al., 2000; Myers et al., 2003; Herzallah et al., 2010; Moustafa and Gluck, 2011). We used it to explore the behavioral and cognitive significance of associative learning in GTC epileptic patients. We represented the connection between hippocampus and basal ganglia as two modules connected to each other by one-to-one connection (see Figure 2).

1. Hippocampus Module: It is a two layered network module consists of 10 patches and 20 nodes, each is considered to be a separate representation code of the input (Grossberg, 1988; Hemmen and Schulten, 1991; Hertz et al., 1991; Kearns and Vazirani, 1994; Miller and MacKay, 1994; Moustafa et al., 2009). We used winner-take-all for simulating the lateral inhibitory connections among neurons in each patch, which are connected to the basal ganglia module (Barto, 1995; Berns and Sejnowski, 1996; Schultz et al., 1997; Suri and Schultz, 1998; Moustafa et al., 2009). Additionally, weight update of the hippocampus was based on soft max probability (see Equations 1.0 and 1.1, Mathematical Appendix).
2. Basal Ganglia Module: It is based on a trial by trial learning rule known as the Rescorla-Wagner rule (Rescorla and Wagner, 1972). This rule relies on a simple linear prediction of the reward associated with a stimulus. We used a binary input variable (*u*) as an indication of the presence or absence of the stimulus (*u* = 1 in case the stimulus is present while *u* = 0 in case of its absence), (see Equations 2 and 3, Mathematical Appendix). The basal ganglia input represents the learning rate value; enabling to assess the association between all the possible input stimuli with the reward (see Equations 3, 4.0, 4.1, 4.2, 5.1, and 5.2, Mathematical Appendix).

#### Model fitting to AEALT

**Figure 3:**
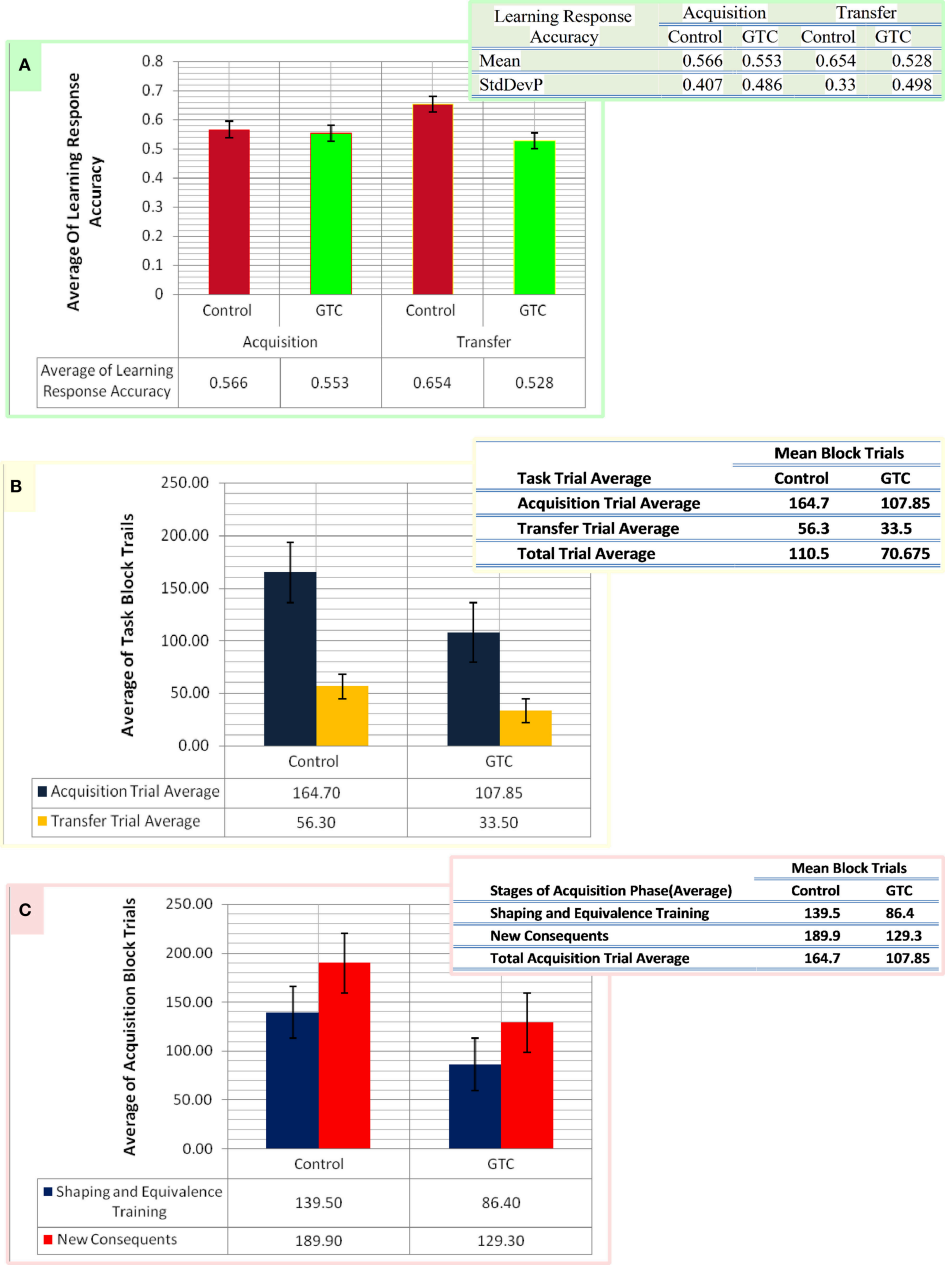
**This figure represents the data of our subjects which considered as an input for our computational model**. Panel **(A)** shows the average of the accurate response in associative learning in GTC epileptic patients and their healthy controls during both phases of AEALT; acquisition and transfer phases of whilst the associated table to **(A)** represents the mean and the standard deviation of the average accuracy responses. **(B)** Represents the average of block trials which were performed by the GTC epileptic patients and their healthy controls during acquisition and transfer phase, whereas these values, which are represented in the associated table to **(B)**, were required as input for our simulated model. **(C)** Indicates the average of block trials which were performed by the GTC epileptic patients and their healthy controls during the stages of shaping and equivalence training, and in the new consequent one, which resemble the acquisition phase, whereas these values, which are shown in the adjacent table to **(C)**, were required as inputs for our simulated model.

We specified the pattern of inputs in term of task stimuli (antecedent and consequent) which were randomly assigned in AEALT (see task description and Figure 1). Therefore, we considered a stochastic policy, which means that when subject used to choose a particular colored fish associated with a specific human face, it would be associated with probabilities of choice either left or right (see Equations 1.0, 1.1, and 3, Mathematical Appendix). We adjusted the values of such probabilities during the associative learning process on the basis of the reward provided for the subject according to the slope of soft max probability (β). For large β; the probability of an action was either raised rapidly to 1 or fallen rapidly to 0, as the difference between the action values increased or decreased, then, the action choice of the subject almost a deterministic choice action. When β is small, the soft max probability is approaching to 1 or 0 more slowly, and the subject's actions are more variable and random. In our model, we adjusted β of soft-max function in each simulation time step of the hippocampus weight update, whereas we represented action selection using soft-max function, which was responsible for hippocampus weight updates. All possible inputs for AEALT (Moustafa et al., 2000; Myers et al., 2003; Herzallah et al., 2010; Moustafa and Gluck, 2011), consists of 16 input from antecedent and consequent stimuli and four inputs from the hippocampus. The 16 inputs represent four different human faces; each one is associated with four different colored fishes, only two are appearing on the screen for each face per trial. For hippocampus inputs; they represent the hippocampus strength having value in the ranges of 0, 1, 2, 3, 4. We considered the direct actor as a simple method for solving the subject' learning response performance in AEALT (Moustafa et al., 2000; Myers et al., 2003; Herzallah et al., 2010; Moustafa and Gluck, 2011), since we focused on static action choice where the reward immediately was followed by the taken action (Montague et al., 1995; Dayan and Abbott, 2001; Chapter 9 Classical Conditioning and Reinforcement Learning; see Equations 3, 4.0, 4.1, and 4.2, Mathematical Appendix). Accordingly, the choice of actions was based directly on maximizing the expected average reward. On the other hand, we represented this reward value using stimulus index associated with choosing actions, where we represented a list of rewards for each stimulus in rows featuring all possible inputs, and columns featuring left or right choosing action. Accordingly, we computed *Q*-values for direct actor for this chosen action. Also, we represented the learning rate value as basal ganglia input and the connection between both modules by one-to-one connection. By the end, we computed the mean and variance of the accuracy, correct performance of the subject. Importantly, we fitted the model with the average of experimental block trials that GTC epileptic and control subjects performed during each phase of AEALT (see Figure 3), before running the simulation for each subject.

#### Mathematical appendix

- **Parameterization of choice probabilities either left or right using softmax distribution:**
  (1.0) $$\begin{array}{l} p[r]=\text{exp}(\beta m_{r})/((exp(\beta m_{r})+(\text{exp}(\beta m_{l})),\\ p[l]=\text{exp}(\beta m_{l})/((\text{exp}(\beta m_{l})+(\text{exp}(\beta m_{r})). \end{array}$$
  Where *P*[*r*] and *P*[*l*] represent the probability of right and left choices, respectively, both probabilities are similar to a sigmoid function of β (*m*_*r*_ − *m*_*l*_) and β (*m*_*l*_ − *m*_*r*_). β represents the slope of soft max function. The parameters *m*_*r*_ and *m*_*l*_ determine the frequency at which corresponding face is chosen. *P*[*r*] + *P*[*l*] = 1, indicates the fact that the model invariably makes one of the two choices, either left or right. The values of *P*[*r*] and *P*[*l*] are adjusted during AEALT on the basis of the reward provided. For large β, the probability of an action rises rapidly to one, or falls rapidly to zero, as the difference between the action values increase or decrease. This makes the chosen action of the subject almost a deterministic function of the *m* variables. If β is small, then, the softmax probability approaches one or zero more slowly, and the action choices of the subject are more variable and random. We adjusted β-value over simulation trials, according to the value of the hippocampus input, which represented the hippocampus strength.
  (1.1) $$\begin{array}{l} P[a]=\text{exp}(\beta m_{a})∕\sum^{2}à=1\;(\beta m_{à}). \end{array}$$
  Where *P*[*a*] is the probability vector *m* of choosing action *a*, which controls the decision process. à = 1, 2 represents the possibility of choices between two actions; right or left.
- **The Rescrola-Wagner learning rule(a version of delta rule):**
  (2) w→w+εδu with δ=r−υ.
  Where *w* represents the weight update, δ represents the prediction error. *U* is a binary variable which indicates the presence or absence of the stimulus, if *u* = 1, then the stimulus is present and *u* = 0 in case of its absence. υ is the expected reward. ε is a coefficient which indicates balancing state of the weight update; if it is sufficiently small, the rule changes *w* systematically until the average value of δ reaches to zero, at this point *w* fluctuates about the equilibrium value in approximation.
- **Direct Actor Equations:**
  (3) $$<r>=P[r]<r_{r}>+P[l]<r_{l}>.$$
  Where < *r*> is the expected average of the reward, *P*[*r*] is the probability of choosing the right choice. < *r*_*r*_> is the average of reward based on choosing the right choice. *P*[*l*] is the probability of choosing the left choice. < *r*_*l*_> is the average of reward based on choosing the left choice. In the Direct Actor method, the choice of actions is based directly on maximizing the expected average of the reward.
  (4.0) $$m_{à}\to m_{à}+\varepsilon (\delta_{aà}-P[à])(r_{a}-\text{ŕ}).$$
  Where *m*_à_ is an action, which is taken for all value of à.
  (4.1) $$m_{à}(u)\to m_{à}(u)+\varepsilon (\delta_{aà}-P[à;u])\delta .\;$$
  Where *u* is a binary stimuli input.
  (4.2) $$m=M.\;u(u)\text{ or }m_{a}=\sum_{b}M_{ar}u_{r}(u).\;$$
  In Equations (4.1) and (4.2); the update of action probability depends on value of δ, when δ > 0 is taken, which increases the probability of the action and when δ < 0 is taken, this value decreases the probability of the action. This means increasing the chance that the subject makes the correct accurate response. Then, the actor learning rule is modified to make use of the information provided by the state vector through generalizing the action from value vector m to action matrix *M*. *M* has as many columns as there are components of *u* (binary stimuli input) and as many rows as there are actions (reward). Given a binary stimuli input *u*, action *a* is chosen at location *u* with the soft max probability of Equations (1.0) and (1.1), using component a of the action value vector.
  These previous equations show how to change the elements of the action matrix (*M*), when action *a* is chosen at location *u* with state vector *u(u)*, leading to location *u*.
- **Policy Actor learning rule equations:**
  (5.1) $$m_{r}\to m_{r}+\varepsilon (\delta_{ar}-P[r])(r_{a}-\text{ŕ}).$$
  (5.2) $$m_{l}\to m_{l}+\varepsilon (\delta_{al}-P[l])(r_{a}-\text{ŕ}).$$
  Where *r*_*a*_ is the selected action; either right or left. δ_*ar*_ and δ_*al*_ are the Kronecker delta, δ_*ar*_ = 1 if *a* = *r* and δ_*ar*_ = 0 if *a* = *l* and δ_*al*_ = *1* if *a* = *l* and δ_*al*_ = 0 if *a* = *l*. ŕ is the mean of the reward under the specified policy. These equations perform the stochastic gradient ascent on the average reward, whatever the value of ŕ (the mean of the reward). Different values of ŕ lead to different variation of the stochastic gradient terms, and thus different speeds of learning.

#### Model implementation

The algorithms had been adjusted from Dayan and Abbott (2001), Chapter 9 Classical Conditioning and Reinforcement Learning whilst the code was implemented using Python (Python Software Foundation. Python Language Reference, version 2.7; van Rossum 1995), BRIAN, neurosimulator based Python Library (Stimberg et al., 2014) and Mat Lab (MATLAB 8.0 and Statistics Toolbox 8.1, The Math Works, Inc., Natick, Massachusetts, United States) softwares.

## Results

### Experimental behavior

Our experimental behavioral results showed no considerable differences in the average of accuracy in learning response performance of GTC epileptic patients and controls during acquisition and transfer phases of AEALT. In the acquisition phase; the average accurate performance of associative learning in GTC was not significantly different (*p* = 0.68, two-tailed test) from controls. Similarly, in the transfer phase the difference was not strong either (*p* = 0.97, two-tailed test) (see Figure 3). These results indicated that AEALT did not reveal cognitive impairment in GTC epileptic patients, neither in hippocampus (associated with the transfer (Tamminga et al., 1992; Buchanan et al., 1993; Bunsey and Eichenbaum, 1995; Henke et al., 1997; Heckers et al., 1999; Myers et al., 2003; Polgár et al., 2010; Moustafa and Gluck, 2011), nor in basal ganglia (associated with the acquisition; Tamminga et al., 1992; Buchanan et al., 1993; Moustafa et al., 2000; Myers et al., 2003; Polgár et al., 2010; Moustafa and Gluck, 2011).

### Computational modeling

**Figure 4:**
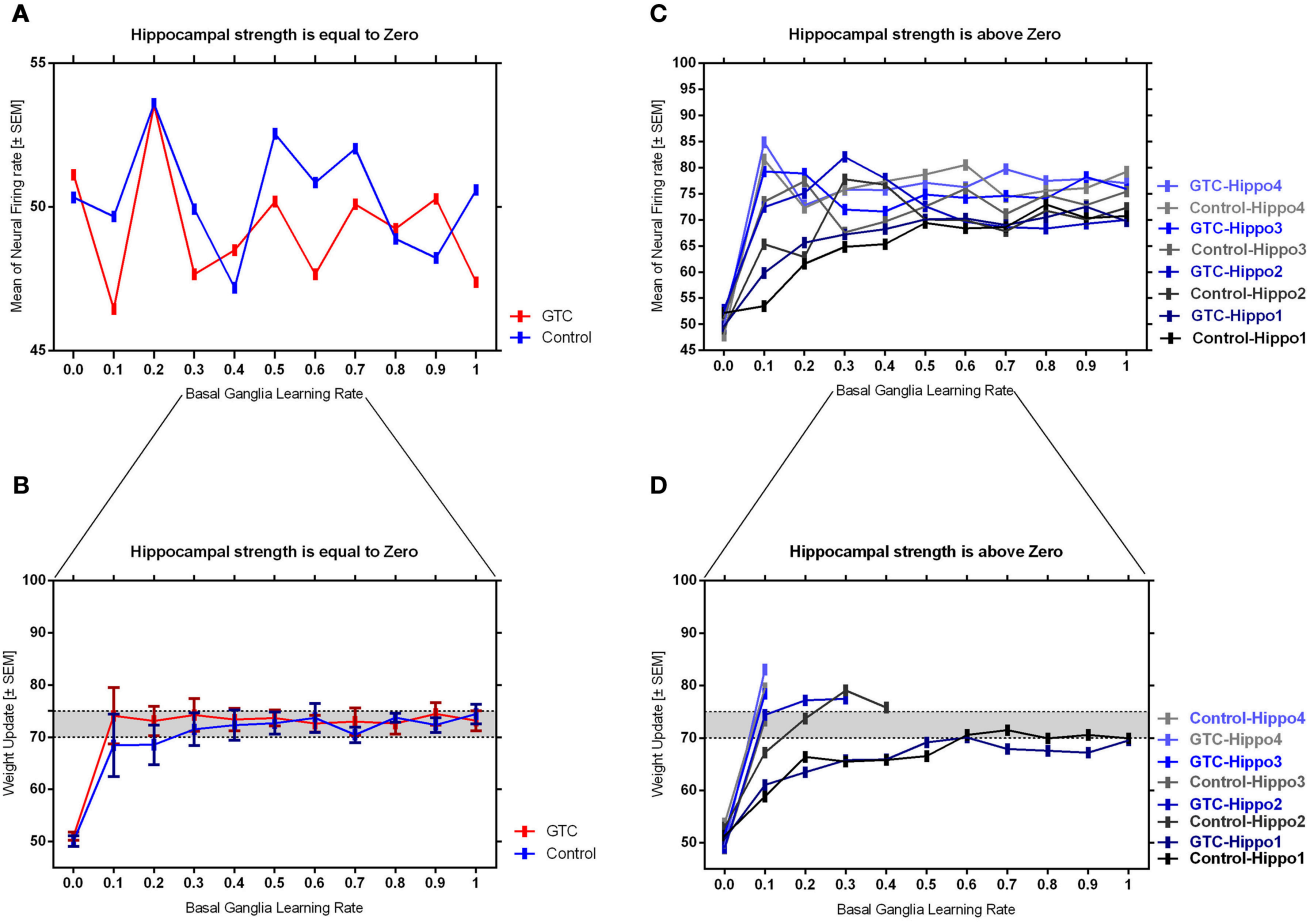
**This figure represents the average of the neural firing rates in response to the connection between basal ganglia and hippocampus when representing different values of hippocampus input indicating hippocampal strength**. Panel **(A)** shows the average of neural firing rates when hippocampal input equal to zero, which triggers the neural firing rate relates to GTC epileptic patients and their controls as an outcome of our simulated model. The X axis represents the learning rate of basal ganglia, as basal ganglia input, and hippocampus input, as hippocampus strength, whereas a zero value of hippocampus input refers to its absence whilst Y axis shows the proportion of the neural firing rate which is in approximation below 55% for both subjects. In GTC-epileptic patients, this firing rate is lower than the controls except when the values of learning rate parameters of the basal ganglia are 0.2 and 0.8 and this rate tends to increase to be higher in GTC than in controls when the learning rate value is 0.9. **(B)** Represents the evaluation of weight update in basal ganglia module when hippocampus strength is absent, as in **(A)**, whereas hippocampus input is equal to zero. This evaluation is represented in terms of percentages referring to GTC epileptic patients and their controls as an outcome of our simulated model. Notably, the percentage of weight update is still below 55% for both GTC epileptic patients and controls, whereas the evaluation of weight update of the basal ganglia relates to the action of the direct actor either right or left. In GTC epileptic patients, the evaluation of weight update of basal ganglia is higher as compared to controls when the learning rate value of basal ganglia is below 0.6. Above 0.6, this evaluation starts to decline to be lower than that of control. Additionally, this evaluation of the weight update is in synchronization with that of controls when the learning rate of basal ganglia is around 0.9. Panel **(C)** shows the average of neural firing rates when hippocampal input is above zero, taking the values of 1, 2, 3, and 4, whereas each value resemble a different state of hippocampus strength which is represented mathematically as input values. Controversially to **(A)**, Hippocampus inputs with values higher than zero refers to the presence of hippocampus with differential strengths. The learning rate values of the basal ganglia were critical for modulating the neural firing rates produced by stabilizing it. However, as long as they increase, the firing rate activities remained comprised between 68 and 81%. **(D)** Represents the evaluation of weight update in basal ganglia module when hippocampus strength, input, is above zero, taking the same values mentioned in **(C)**. In general, the evaluation of weight update of the basal ganglia increased rapidly when the hippocampus strength was large (values 3 and 4) while it slowly increased when the hippocampus strength was less (values 1 and 2).

Our abstract model-based analysis of AEALT (Moustafa et al., 2000; Myers et al., 2003; Herzallah et al., 2010; Moustafa and Gluck, 2011), showed a strong connection between hippocampus and basal ganglia in GTC epileptic patients. This connection was represented in our connectionist model by two modules (hippocampus and basal ganglia), which connected to each other by one-to-one connection. We introduced different values of hippocampus input representing differential states hippocampus strength. Then, we associated each of these values with different values of the basal ganglia input representing the learning rate values. In our simulation, we considered two different conditions based on the strength of hippocampus (hippocampus input) while fixing the values of basal ganglia input (the learning rate values) to have range of values in each condition between 0, 0.1, 0.2, 0.3, 0.4, 0.5, 0.6, 0.7, 0.8, 0.9, and 1. In each condition, we measured the changes in the neural firing rate and observed the evaluation of the basal ganglia weight update in response to the changes in the inputs of hippocampus and basal ganglia. For the first condition, the hippocampus strength was equal to zero while the second one represented it to be above zero, with values ranging between 1, 2, 3, and 4. In the first condition, we observed that the percentage of the neural firing rate in GTC epileptic patients and controls was below 55%. Additionally, the weight update evaluation of the basal ganglia was in the same range as the neural firing rate (below 55%). Thus, the learning rate of basal ganglia had an impact on scaling the average of neural firing rate and also a robust role in guiding the choosing action either left or right when hippocampus strength was zero (see Figure 4 and Table 1).

**Table 1:** **It represents the values of the neural firing rate in GTC epileptic patients and their controls in our connectionist simulated model which are shown in Figure 4**.

**Learning rate values**			**0**	**0.1**	**02**	**0.3**	**0.4**	**0.5**	**0.6**	**0.7**	**0.8**	**0.9**	**1.0**
**(A) Mean firing rate values**													
Control			50.33	49.67	53.59	49.93	47.19	52.55	50.85	52.03	48.89	48.23	50.59
GTC			51.12	46.45	53.55	47.66	48.50	50.19	47.66	50.09	49.25	50.28	47.38
**(B) Mean weight update values**													
Control			50.85	48.13	48.37	50.85	50.2	49.15	53.46	46.41	49.54	48.89	52.94
GTC			50.37	51.21	50.28	50.37	50.8	52.24	49.99	48.04	47.85	49.25	52.15
**(C) Mean firing rate values**													
Control	Hippocampus strength	1	52.17	53.46	61.57	64.84	65.36	69.41	68.37	68.6	72.94	70.33	70.72
GTC		1	49.63	59.81	65.61	67.2	68.22	70.09	70.28	69	70.47	72.52	69.72
Control		2	49.15	65.36	62.88	77.78	76.73	70.06	69.93	67.8	71.76	70.06	72.29
GTC		2	52.8	72.43	75.14	82.06	77.94	72.62	69.63	68.6	68.32	69.25	69.99
Control		3	51.24	73.46	77.39	67.58	69.67	72.55	75.95	71.1	74.77	72.81	75.42
GTC		3	51.87	79.25	78.88	71.96	71.59	74.86	74.21	74.6	74.11	78.22	75.89
Control		4	47.84	81.57	72.29	75.82	77.38	78.69	80.52	74.3	75.55	76.08	79.22
GTC		4	49.81	84.86	72.8	75.79	75.7	77.1	76.26	79.7	77.48	77.85	77.01
**(D) Mean weight update values**													
Control	Hippocampus strength	1	51.37	58.82	66.4	65.49	65.8	66.54	70.59	71.5	69.93	70.59	69.93
GTC		1	50.75	61.03	63.46	65.79	65.9	69.16	70.09	67.9	67.57	67.2	69.53
Control		2	52.94	67.19	73.72	79.08	75.8						
GTC		2	49.06	74.39	77.2	77.48							
Control		3	48.89	73.33									
GTC		3	51.59	78.41									
Control		4	53.72	79.48									
GTC		4	49.53	83.08									

*Panel **(A)** is linked to Figure 4A whilst panels **(B–D)** are associated to Figures 4B–D, respectively*.

In the second condition, we observed that the percentage of the neural firing rate in our subjects was increasing to be above 50%. Also, the weight update evaluation of the basal ganglia was in the same range as the neural firing rate in this condition (above 50%), (see Figure 4 and Table 1), which means that the neural firing rate was increasing with increasing hippocampus strength (hippocampus input). GTC epileptic patients showed less neural firing rate as compared to controls in each state of hippocampus strength (i.e.; different hippocampus input values). Additionally, changes in such neural firing rate were sensitive to the changes in the basal ganglia inputs. All together indicated that the learning rate values of the basal ganglia were critical for modulating the neural firing rate of the connectionist model by stabilizing it as long as its' increase remained comprised between 68 and 81%. In general, there was a reasonable correlation between the percentage of the neural firing rate (see Figure 4 and Table 1), and evaluation of weight update in GTC epileptic patients and controls in each condition. For example, when hippocampus strength was above zero, the percentage of weight update was above 50% for both GTC epileptic patients and controls. Moreover, the weight update of the basal ganglia was increasing with increasing in the hippocampus strength, i.e.; the weight update of the basal ganglia was very sensitive to hippocampus strength. For example, the evaluation of weight update of the basal ganglia was increasing rapidly when the hippocampus strength was large (values 3 and 4) while this increase was slow when the hippocampus strength was less (values 1 and 2). This observation suggested a strong influence of the hippocampus strength on weight update evaluation of the basal ganglia and hence strong connection between them.

## Discussion

To our knowledge, this present study is the first to assess AEALT (Moustafa et al., 2000; Myers et al., 2003; Herzallah et al., 2010; Moustafa and Gluck, 2011), in patients with the GTC epilepsy. We measured the accuracy of associative learning in GTC epileptic patients through AEALT. We relied on using combined experimental behavioral and computational study to link our experimental findings with associative acquired equivalence principles; neurobiological, psychologically and theoretically. One clear limitation of our study is the low number of GTC patients and their healthy subjects that was studied. Therefore, our results will need to be confirmed by further complementary studies at larger scale in the near future. However, our approach allowed to design a computational model that can be fed from actual subjects' data. Therefore, this model will be useful to test the role of the connectivity between the frontal and temporal lobes in cognitive functions, and its possible alterations in epilepsy. Importantly, most of the studies dealing with epilepsy used a low number of subjects due to the difficulty to recruit large populations, in particular in a case of cognitive and behavioral studies. Even within the largest postoperative cognitive study in adult, Helmstaedter and Witt (2008) included only 39 patients and the largest pediatric study of Gleissner included only 15 patients (Gleissner et al., 2008). Even the study with the most comprehensive neuropsychological testing and the largest sample (*n* = 11) (Picard and Craig, 2009) had only 5 patients in the sample having IEDs on EEG, and the IEDs. In addition, these studies are composed of highly heterogeneous groups of patients, including wide age ranges, divergent seizure characteristics and, in some cases, even different surgical procedures (e.g., cortical reactions, lesions and/or multiple transactions). In order to be more consistent, we paid a special attention to investigate homogenous group made of healthy subjects and matched GTC patients, that is of patients suffering from a specific type of epilepsy. Due to the limited size of our sample groups, it was difficult to draw a robust statistical significance from our results, whereas; we focused on testing the validation of this connectionist model through using our patients' data as an input for the model. Our experimental behavioral results showed that the accuracy of learning performance was not different in GTC patients as compared to controls (see Figure 3). This result indicated no impairments neither in hippocampus (associated to transfer phase; Tamminga et al., 1992; Buchanan et al., 1993; Bunsey and Eichenbaum, 1995; Henke et al., 1997; Heckers et al., 1999; Myers et al., 2003; Polgár et al., 2010; Moustafa and Gluck, 2011), nor in basal ganglia (associated with acquisition phase; Tamminga et al., 1992; Buchanan et al., 1993; Moustafa et al., 2000; Myers et al., 2003; Polgár et al., 2010; Moustafa and Gluck, 2011). On the other hand, our computational simulation findings confirmed our experimental behavioral results since they showed strong connection between hippocampus and basal ganglia modules. We segregated our simulation into two conditions based on the strength of the hippocampus (hippocampus input); either equal or above zero. In each condition, we fixed the values of the basal ganglia inputs, which represented the learning rate values, to be in the ranges of 0, 0.1, 0.2, 0.3, 0.4, 0.5, 0.6, 0.7, 0.8, 0.9, and 1. Then, we measured the average of the neural firing rate and weight update evaluation of the basal ganglia in response to the changes of the input of the hippocampus referring to hippocampus strength. In the first condition, when hippocampus strength was equal to zero, the average of the neural firing rate in both GTC and controls was below 55%. In addition, the weight update evaluation of the basal ganglia was similar to this range (below 55%); this observation indicated the robust role of the basal ganglia input in guiding the direct actor either left or right. All together approved the considerable impact of hippocampus strength, hippocampus input, and the learning rate of basal ganglia, basal ganglia input, on modulating of the neural firing rate of our connectionist model (see Figures 4A,B and Table 1). On the other hand, in the second condition, when hippocampus strength was above zero, taking ranges of 1, 2, 3, and 4, the average of the neural firing rate in GTC and controls was above 50% and the weight update evaluation of basal ganglia was in line with this range of the neural firing rate (above 50%), (see Figures 4C,D and Table 1). In this case, changes in the basal ganglia inputs were sensitive to the changes in the hippocampus input (differential state of hippocampus strength). However, this sensitivity was observed only when hippocampus strength had a value of 1, with increasing hippocampus inputs; the influence of basal ganglia inputs was decreasing. For example, the average of the neural firing rate reached to the highest peak when hippocampus input was in the range of 4 and 3 although the value of basal ganglia input was very small or nearly absent (around 0–0.1). Then, there was no need for increasing the value of basal ganglia input above 0.1 to enhance the neural firing rate of our model. This observation indicated that learning rate values of basal ganglia had a role in modulating and stabilizing the mean neural firing rate of the model only when hippocampus strength was around 1. Additionally, weight update of the basal ganglia increased rapidly and immediately when hippocampus strength was larger (values 3 and 4) while it increased slowly and regularly when hippocampus strength was smaller (values 1 and 2). Generally, GTC epileptic patients showed less neural firing rate as compared to controls in each state of hippocampus strength. In conclusion, the two conditions that we explored in our study explained how the interactive connection between basal ganglia and hippocampus module influenced the neural activity after changing the learning rate of both, the basal ganglia, representing in basal ganglia input, and the hippocampus strength, which resembles hippocampus input. We did not include Temporal Difference (TD) learning Algorithm (Dayan and Abbott, 2001; Chapter 9 Classical Conditioning and Reinforcement Learning) to measure the temporal differences in GTC epileptic patients within both phases of acquired equivalence task; acquisition and transfer. Alternatively, We relied on using direct actor method and Rescorla Wagner rule instead of TD since we preferred to use this simple method in the beginning of our study with GTC epileptic patients, aiming to extend this study with GTC epileptic patients further in the future studies. While cognitive impairments, and especially memory disruption (Henke et al., 1997), are prominent comorbidity in patients with epilepsy, their path physiology remains unknown (Bell et al., 2011). Recent studies concluded that cognitive impairment in epilepsy results from a network disorder in which the micro-structures as well as the functionality can be disturbed (Braakman et al., 2012). In this present study, however, we did not find any considerable difference in associative learning between GTC patients and control subjects. This lack of impairment may be due to several factors. First, the number of our subjects remained small and the study should be extended to a larger number of subjects, which we are planning to do in the future studies. No doubt, including more GTC epileptic patients with different ages and/or sex will enable us to detect possible correlation between ages and/or sex in shaping the acquired learning performance in GTC epileptic patients. Second, the age of the patients seems to be a critical factor. Indeed, a very recent study found no difference in neuropsychological performances in children with temporal lobe epilepsy (Mankinen et al., 2014) whilst a study with animal models has shown that early-life seizures are key events that contribute to deficits in learning (Lugo et al., 2014). In this current study, our patients did not experience a long history of seizures, which can explain the absence of associative learning impairment. Despite lack of differences with AEALT (Moustafa et al., 2000; Myers et al., 2003; Herzallah et al., 2010; Myers et al., 2003), the GTC patients may show fine structural and/or functional alterations of the brain networks, which could be involved, besides, the relatively small sample size examined here may limit the representativeness of patients with GTC. Therefore, additional numbers of patients with GTC will be added in further future studies as a continuation of the present work. Moreover, to address this question further, we will need to complement these behavioral and model studies with imaging studies aiming to describe the functional connectivity between basal ganglia and hippocampus in the future. Regarding the methodological aspect of our study, independently of the functional result, our main aim was to validate a modeling approach. Importantly, our simulation protocol proved a reasonable efficiency to reproduce the results obtained with a cognitive behavioral task. In summary, the main result of the present work was to provide a simulation method that permits to analyses functionally the network (i.e., basal ganglia and hippocampus) underlying cognitive processes within the context of a neurological pathology.

### Conflict of interest statement

The Review Editor Marc Landry declares that, despite being affiliated to the same institution as author Radwa Khalil, the review process was handled objectively and no conflict of interest exists. The authors declare that the research was conducted in the absence of any commercial or financial relationships that could be construed as a potential conflict of interest.
